# Key enzymes involved in the utilization of fatty acids by *Saccharomyces cerevisiae*: a review

**DOI:** 10.3389/fmicb.2023.1294182

**Published:** 2024-01-11

**Authors:** Zhaoyun Wang, Chunli Su, Yisang Zhang, Sifan Shangguan, Ruiming Wang, Jing Su

**Affiliations:** ^1^State Key Laboratory of Biobased Material and Green Papermaking (LBMP), Qilu University of Technology, Jinan, Shandong, China; ^2^Key Laboratory of Shandong Microbial Engineering, Qilu University of Technology (Shandong Academy of Sciences), Jinan, Shandong, China

**Keywords:** β-oxidation, lipid metabolism, *Saccharomyces cerevisiae*, fatty acids, fatty acyl-CoA, yeast

## Abstract

*Saccharomyces cerevisiae* is a eukaryotic organism with a clear genetic background and mature gene operating system; in addition, it exhibits environmental tolerance. Therefore, *S. cerevisiae* is one of the most commonly used organisms for the synthesis of biological chemicals. The investigation of fatty acid catabolism in *S. cerevisiae* is crucial for the synthesis and accumulation of fatty acids and their derivatives, with β-oxidation being the predominant pathway responsible for fatty acid metabolism in this organism, occurring primarily within peroxisomes. The latest research has revealed distinct variations in β-oxidation among different fatty acids, primarily attributed to substrate preferences and disparities in the metabolic regulation of key enzymes involved in the *S. cerevisiae* fatty acid metabolic pathway. The synthesis of lipids, on the other hand, represents another crucial metabolic pathway for fatty acids. The present paper provides a comprehensive review of recent research on the key factors influencing the efficiency of fatty acid utilization, encompassing β-oxidation and lipid synthesis pathways. Additionally, we discuss various approaches for modifying β-oxidation to enhance the synthesis of fatty acids and their derivatives in *S. cerevisiae*, aiming to offer theoretical support and serve as a valuable reference for future studies.

## 1 Introduction

Fatty acids, a class of alkane compounds with carboxyl groups, are widely used in the chemical, medicine, food, and agricultural sectors. Conventional fatty acids are primarily prepared by cracking animal and vegetable oils and petroleum extracts, and by olefin oxidation. In recent years, owing to the shortage of petroleum resources and the rising prices of petrochemical products (Röttig et al., 2010), the synthesis of fatty acids and their derivatives using microorganisms has gained significant interest (Peralta-Yahya et al., 2012).

The whole genome sequencing and phenotypic analysis of *S. cerevisiae* are more comprehensive compared to those of oil-producing yeast strains. Moreover, as a food-safe strain (Son et al., 2023), it plays a pivotal role as a chassis organism for the efficient production of fatty acids and their derivatives.

The synthesis of fatty acids occurs when glucose is sufficient, and acetyl-CoA synthesizes fatty acids under the action of fatty acid synthase, forming related lipid derivatives in the endoplasmic reticulum and cytoplasm (Gajewski et al., 2017). Under the condition of a low-carbon source, β-oxidation is the only fatty acid degradation pathway can provide energy in *S. cerevisiae*. Long-chain fatty acids (LCFA) are activated by the fatty acyl-CoA synthetase of *S. cerevisiae*, then transported to the peroxisomes through the ABC transporter for β-oxidation (Ferreira et al., 2018). Medium-chain fatty acids are activated by Faa2p and pex11p, located on peroxisome membranes, as medium-chain lipoyl-CoA, and transported into peroxisomes for β oxidation.

Saturated fatty acids can be directly metabolized via β-oxidation, whereas unsaturated fatty acids must first be converted to trans-dienoyl-CoA by auxiliary enzymes before entering the β-oxidation pathway (Yocum et al., 2022). A β-oxidation cycle is the process of releasing an acetyl-CoA molecule from the carboxyl terminus of a fatty acid. Fatty acids are first activated by fatty acyl-CoA synthetase to form fatty acyl-CoA, and then dehydrogenated by fatty acyl-CoA oxidase to form trans-2-enoyl-CoA. Trans-2-enoyl-CoA is hydrated and dehydrogenated by the multifunctional enzyme Fox2 to form 3-ketoyl-CoA. Finally, 3-ketoacyl-CoA removes one molecule of acetyl-CoA under the action of 3-ketoyl-CoA thiolase Pot1p, and then proceeds to the next β-oxidation cycle. With the release of acetyl-CoA, fatty acids are eventually degraded into acetyl-CoA, which is then transported to the cytoplasm or mitochondria to be used in other biological pathways. However, in addition to the key enzymes mentioned above, the β-oxidation process requires cofactors such as CoA, NADPH, NADH, and ATP.

**Figure 1:**
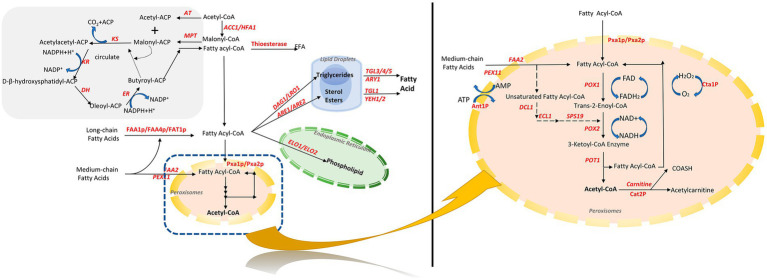
Fatty acid synthesis and metabolic pathways in *S. cerevisiae*. Orange is peroxisomes, green is endoplasmic reticulum, and blue is lipid droplets. Acetyl-CoA and malonic acid monoacyl-CoA synthesize fatty acids in the cytosol through fatty acid synthase. Fatty acids are activated in the cytosol as fatty acyl-CoA, followed by the synthesis of phospholipids by elongation enzymes in the endoplasmic reticulum, and triglycerides and sterol esters in the cytosol, which are located inside the sterol esters, which are in the center of the lipid droplets. Exogenous and endogenous fatty acids are finally degraded to acetyl-CoA by a unique β-oxidation pathway within *S. cerevisiae*. PGI, phosphoglucose isomerase; PDC, pyruvate decarboxylase; ALD, acetaldehyde dehydrogenase; Acs, acetyl-CoA synthetase; ACC1, acetyl CoA carboxylase; FAS, fatty acid synthase.

Fatty acyl-CoA is a key intermediate in fatty acid metabolism and transformation in *S. cerevisiae*. In addition to being degraded into acetyl-CoA during β-oxidation, it is an essential intermediate for the synthesis of fatty acid derivatives, such as storage lipids and membrane esters (Figure 1).

The intracellular metabolism of fatty acids plays a crucial role in the accumulation of fatty acids, and investigating the functionality and regulatory expression of enzymes involved in key metabolic pathways is beneficial for controlling fatty acid production in engineered strains. The construction of the reverse pathway for fatty acid β-oxidation is currently a prominent area of research in synthetic fatty acid pathways, and studying the substrate preferences and functional properties of key enzyme catalysis within the β-oxidation pathway is helpful to enrich the catalytic element library of the β-reversal pathway.

## 2 Key enzymes of the β-oxidation pathway

### 2.1 Fatty acyl-CoA synthetase

Studies have shown that a variety of ester acyl-CoA synthases (EC 6.2.1.3) are present in *S. cerevisiae*, including Faa1-4p and Fat1-2p (Watkins and Ellis, 2012), which are localized in the endoplasmic reticulum, lipid granules, plasma membrane, vacuolar membrane, cell periphery, and peroxisomes (Black and DiRusso, 2007). They play a key role in the transportation and activation of fatty acids and have certain preferences for fatty acids of different lengths (Ullah et al., 2021), there are similarities between oil-producing yeast and *S. cerevisiae* in fatty acid metabolism, as shown in Table 1.

**Table 1:** *Saccharomyces cerevisiae* vs. *Yarrowia lipolytica* β-oxidation-related enzymes.

Enzyme	Key features	Location in the cell	Source	Genes/enzymes	Preference for FA chain length	References
Fatty acid synthase (EC 2.3.1.86)	Biosynthesis of fatty acids from acetyl-CoA	Cytoplasmic matrix, mitochondria	Yarrowia lipolytica *S. cerevisiae*	FAS1/2 FAS1/2	C16,C18	Pozdniakova et al. (2023)
Fatty acyl-CoA synthetase (EC 6.2.1.3)	Free fatty acids are activated as fatty acyl-CoA and transported to peroxisomes	Cytoplasmic matrix peroxisomes	Yarrowia lipolytica	ASCI ASCII	C12-C22 C4-C8 C12-C22	Zhang et al. (2022) Liu et al. (2015) Beopoulos et al. (2009)
	Free fatty acids are activated as fatty acyl-CoA in peroxisomes					
	Activation of free fatty acids to fatty acyl-CoA	Endoplasmic reticulum, lipid granules, peroxisomes	*S. cerevisiae*	Faa1p Faa2p Faa4p Fat1p	C10-C18 C6-C12 C14-C18 C20-C26	Melton et al. (2013) Choi and Martin (1999) Knoll et al. (1994)
acyl-coenzyme A oxidase (EC 1.3.3.6)	The oxidation of fatty acids to trans-2-enoyl-CoA is catalyzed in peroxisomes, which oxidizes fatty acids to trans-2-enoyl-CoA	Peroxisomes Peroxisomes	Yarrowia lipolytica	Acox1p Acox2p Acox3p Acox4p Acox5p Acox6p	Dicarboxylic acids C8-C18 C4-C18 C6-C16 C12-C22 Dicarboxylic acids	Wang et al. (1998) Kim and Kim (2018) Ibrahim et al. (2022) Mlíčková et al. (2004) Schäfer et al. (2004)
			*S. cerevisiae*	Fox1p	C8-C12	
hydroxyacyl-CoA dehydrogenase [Includes: D-3-hydroxyacyl-CoA dehydratase (EC 4.2.1.-); 3-hydroxyacyl-CoA dehydrogenase (EC 1.1.1.35)]	Involved in the β oxidation process of fatty acids, trans-2-enoyl-CoA becomes 3-ketoyl-CoA	Peroxisomes	Yarrowia lipolytica	MFE-2	C12-C16	Han et al. (2017) Otzen et al. (2013) Fickers et al. (2011) Bergenholm et al. (2018)
		Peroxisomes	*S. cerevisiae*	Fox2p	Non-specific	
3-ketoacyl-CoA thiolase (EC 2.2.1.16)	3-ketoyl-CoA is broken down into two short carbon chains, fatty acyl-CoA, and one molecule of acetyl-CoA	Mitochondria	Yarrowia lipolytica	Pot1p	C8,C10,C12,C14	van Roermund et al. (2022) Huang et al. (2022) Hanko et al. (2018)
		Mitochondrial membrane space and peroxisome matrix	*S. cerevisiae*	Pot1p	Non-specific	
Adenine nucleotide transporters	Provides ATP when peroxisome acyl-CoA synthetase II activates fatty acids	Peroxisome membrane	Yarrowia lipolytica *S. cerevisiae*	Ant1p Ant1p	Non-specific C8-C12	Plett et al. (2020)
ABC transporters	The long-chain fatty acyl-CoA located in the cytosol Transport to peroxisomes	Peroxisome membrane	Yarrowia lipolytica *S. cerevisiae*	Pxa1p/Pxa2p Pxa1p/Pxa2p	C14-C16	Thevenieau et al. (2007)
Peroxisomal membrane protein 11	Assists Faa2p in transporting medium-chain fatty acids into peroxisomes	Peroxisome membrane	Yarrowia lipolytica *S. cerevisiae*	Pex11p Pex11p	C8-C12 C8-C12	Sartaj et al. (2023)
Delta3-Delta2-enoyl-CoA isomerase (EC 5.3.3.8)	Convert 3-enoyl-CoA into trans-2-enoyl-CoA.	Peroxisome matrix	Yarrowia lipolytica *S. cerevisiae*	Eci1p Eci1p		Mursula et al. (2004)
Peroxisomal acyl-coenzyme A thioester hydrolase 1 (EC 3.1.2.2)	Peroxisomal long-chain acyl-coA thioesterase 1	Peroxisome matrix	Yarrowia lipolytica *S. cerevisiae*	Pte1p Pte1p	C12–C16	Henritzi et al. (2018) Carrier et al. (2019)

Faa1p is an important protein involved in the transport and activation of fatty acids (Li et al., 2007) and is mainly located in lipid granules, the plasma membrane, the endoplasmic reticulum, and mitochondria (Hu et al., 2019; Zhu et al., 2019). Studies have shown that Faa1p plays a key role in the metabolism of long-chain fatty acids (Zhou et al., 2016). Three fatty acyl-CoA (Faa1p-6xHis, Faa2p-6xHis, and Faa3p-6xhis) were isolated and purified from *S. cerevisiae* at 25°C and pH 7.1 by affinity chromatography. Analysis of the activity of the three ester acyl-CoA synthetases on different saturated fatty acid carbon chains (C3–C24) showed that Faa1p has a wide range of substrate catalytic activities on C10–C18 fatty acids, with its most suitable substrate being pentadecanoic acid (Knoll et al., 1994).

Faa4p, which is localized in the endoplasmic reticulum and lipid droplets (Gaspar et al., 2022), acts on long-chain fatty acids (Black and DiRusso, 2007) and has high catalytic effects on C14–C18 fatty acids (Melton et al., 2013). In another study, after using fluorescence-labeled oleate as substrate at a final concentration of 100 μM for 10 min, the intracellular oleyl-CoA concentration in wild type *S. cerevisiae* increased by almost six fold, while that in the Δ*FAA4* strain decreased to approximately 50% of that in the wild type strain (Færgeman et al., 2001). Faa1p and Faa4p play major roles in activating intracellular C14 and C16 saturated fatty acids in *S. cerevisiae* (Knoll et al., 1995). Knockout of *FAA1* or *FAA4* alone did not effectively reduce the activation and consumption of intracellular fatty acids, whereas their combined knockout significantly reduced the activation of cytoplasmic fatty acids (Scharnewski et al., 2008).

Fat1p, an ultra-long-chain fatty acyl-CoA synthetase located in the endoplasmic reticulum, is mainly responsible for the transport and activation of C20–C26 ultra long-chain fatty acids. The activation of 90% of C24 fatty acids in *S. cerevisiae* cells is completed by Fat1p, and *FAT1* knockdown hinders the consumption of ultra long-chain fatty acids (Choi and Martin, 1999). Fat1p and Faa1p or Faa4p are required for the transport of long-chain fatty acid in *S. cerevisiae* (Zou et al., 2003). When *FAA1*, *FAA4*, and *FAT1*, the three synthase genes that are mainly responsible for activating fatty acids, are knocked out at the same time, most of the free fatty acids in the cytosol cannot be activated into fatty acyl-CoA, which means that they cannot be transported to the peroxisomes for β-oxidative degradation, and the fatty acids in the cytosol are accumulated, reaching 490 mg/L (Leber et al., 2015).

Faa2p is located in the peroxisome membrane (Poudyal and Paul, 2022) and its catalytic center is situated at its C-end site. It belongs to a class of fatty acyl-CoA synthetases (Van Roermund et al., 2001) reported to have a predilection for medium-chain fatty acids (C6–C12 or C9–C13; Knoll et al., 1994). *S. cerevisiae* lacking Faa2p cannot effectively utilize medium-chain fatty acids, such as caprylic acid, decanoic acid, and dodecanoic acid, via β-oxidation (Zou et al., 2003). It has been reported that Faa2p acts synergistically with Fat1p in the activation of ultra long-chain fatty acids (Falcon et al., 2010). After single knockout of *FAT1*, oleic acid induces Faa2p expression in peroxisomes, which leads to ultra-long chain synthase activity. After continuing to knock out the *FAA2* gene, the residual ultra-long chain acyl-CoA activity in strains that double-knocked out *FAT1* and *FAA2* genes was less than 5% of wild-type strains and less than 20% of single-knockout *FAT1* strains (Choi and Martin, 1999).

Only a few studies have been published on the function of Faa3p. Through the induction of *FAA3* overexpression in *Escherichia coli*, Faa3p was found to have catalytic activity on 9-hexadecanoic acid and 9-ocadecanoic acid (Knoll et al., 1994). The activation of fatty acid transport in *S. cerevisiae*, as well as the activation of the five ester acyl CoA synthetase genes, *FAA1*, *FAA2*, *FAA4*, *FAT1*, and *FAT2*, involve some unidentified proteins (Van Roermund et al., 2000). In a previous study, the acyl-CoA synthetases genes *FAA1* and *FAA4* were completely knocked out, radiolabeled oleic acid was added to the medium, and gas chromatography analysis revealed that Faa1p and Faa4p were mainly responsible for the utilization of intracellular fatty acids (Scharnewski et al., 2008). Compared with *S. cerevisiae*, *Yarrowia lipolytica* fatty acyl-CoA synthetase has a transport function in addition to the role of activating fatty acids and the intracellular Long-chain fatty acids is activated to fatty acyl-CoA by fatty acyl-CoA synthetase ACS I. Long chain fatty acyl-coA is degraded by the transfer protein into the peroxisome, while medium and short chain fatty acids enter the peroxisome directly to be activated by ACSII and then undergo β-oxidation (Zhang et al., 2022).

### 2.2 Fatty acyl-CoA oxidase (*FOX1*)

Fox1p (EC 1.3.3.6) is the second key enzyme in the *S. cerevisiae* β-oxidation pathway; it is located in the peroxisome matrix and oxidizes acyl-CoA to trans-2,3-enoyl-CoA (Han et al., 2017). Following *FOX1* knockout, *S. cerevisiae* was found not to grow normally on a medium with oleic acid as the sole carbon source (Lefevre et al., 2013), suggesting that Fox1p is the only fatty acyl-CoA oxidase in *S. cerevisiae*. *FOX1* transcription is regulated by intracellular glucose, ethanol, and long-chain fatty acid levels (Wang et al., 1994). No *FOX1* transcription was detected when the glucose concentration in the medium was >0.1% (Wang et al., 1992). However, when ethanol was used as the sole carbon source for culturing, a small amount of *FOX1* mRNA in the stable state was detected. Furthermore, when the medium contained only oleic acid, higher *FOX1* transcription levels were detected, with its mRNA levels being approximately 25 and 10 times higher than that in the glucose- and ethanol-containing media, respectively. Moreover, it has been reported that *FOX1* transcription is strictly regulated by the carbon source (Stanway et al., 1995).

Oleogenic yeast cells often contain multiple fatty acyl-CoA oxidase genes. For example, *Yarrowia lipolytica* contains six fatty acyl-CoA oxidase genes (ACOX1–6). Acox1p is inactive, and in its presence alone, the strain cannot grow on a medium with oleic acid as the sole carbon source (Luo et al., 2002). Acox2p oxidizes medium- and long-chain acyl-CoAs and has good catalytic activity on C10–C14 straight chain acyl-CoAs (Kim and Kim, 2018). Acox3p preferentially oxidizes short-chain acyl-CoA, and *Yarrowia lipolytica* cells lacking Acox2p and Acox3p exhibited normal growth on a medium with glucose; however, the activity of intracellular fatty acyl-CoA oxidase was only one-fifth the total oxidase activity. This suggests that Acox2p and Acox3p are important acyl-CoA oxidases in *Y. lipolytica* (Mlíčková et al., 2004). Acox4p exhibits catalytic activity on both medium- and long-chain fatty acids (C6–C16), but has a predilection for long-chain fatty acids; in addition, Acox5p exhibits significant catalytic activity on fatty acids longer than 12 carbon atoms (Hilmi Ibrahim et al., 2020). Three fatty acyl-CoA oxidases, Pox2p, Pox4p, and Pox5p, have been identified in *Candida tropicalis*; Pox4p is a long-chain fatty acyl-CoA oxidase (Cao et al., 2017). Plant cells also typically contain several acyl-CoA oxidase isoenzymes, which are specific to long, medium, and short-chain fatty acids depending on the structural function of the enzyme (Zhang et al., 2017).

### 2.3 Hydroxyl-CoA dehydrogenase/enoyl-CoA hydratase (*FOX2*)

Fox2p [Includes: D-3-hydroxyacyl-CoA dehydratase (EC 4.2.1.-); 3-hydroxyacyl-CoA dehydrogenase (EC 1.1.1.35)] is a multifunctional *S. cerevisiae* β-oxidation enzyme with both enoyl-CoA hydratase and hydroxyl-CoA dehydrogenase activity; it catalyzes the transformation of trans-2,3-enoyl-CoA to 3-ketoacyl-CoA (Knoblach and Rachubinski, 2018). Researchers have attempted to induce *FOX2* expression in the cytoplasm of *S. cerevisiae*; however, no enzyme activity was detecte, probably because *FOX2* cannot fold into the correct conformation in the cytoplasm of *S. cerevisiae* (Lian and Zhao, 2015).

*FOX2* transcription also requires oleic acid induction (Fickers et al., 2011; Darvishi, 2012) and is regulated by the Pip2-Oaf1 complex (Bergenholm et al., 2018). The oleic acid-activated transcription factor, Pip2p, which is a subunit of the Pip2-Oaf1 complex, contains a typical Zn (2)-Cys (6) cluster domain. In the absence of glucose in the culture medium but with sufficient oleic acid, both Pip2 and Oaf1 bind to OREs in the promoter region of the gene that encodes the peroxisome protein (Otzen et al., 2013) to form a heterodimer (Han et al., 2017) that positively regulates the transcription of genes involved in fatty acid β-oxidation and that of RNA polymerase II (Gurvitz et al., 1999). Rottensteiner et al. (1996) found that strains with *PIP2* deletion exhibited impaired growth, could not transcribe β-oxidation-related enzymes, and had reduced peroxisome number and size in a medium with oleate as the sole carbon source. These results indicate that *PIP2* and *OAF1* are essential genes for fatty acid-induced peroxisome proliferation and the regulation of fatty acid oxidation; these findings also indicate that fatty acids can activate *PIP2*, thereby improving the transcription of β-oxidation-related genes.

### 2.4 3-ketoacyl-CoA thiolases (*POT1*)

Only one thiolase gene, *POT1*, has been identified in the *S. cerevisiae* genome (Huang et al., 2022). Mercaptase, which plays an important role in lipid metabolism, is localized in the mitochondrial membrane space and peroxisomal matrix as a homologous dimer. The sequences of the first 16 amino acids at its N-terminus are the same as the localization sequences of thiolases (Huang et al., 2022).

Studies on the crystal structure of *S. cerevisiae* peroxisome thiolases have revealed their binding pattern to substrate molecules and their reaction mechanisms (Mathieu et al., 1997). Pot1p (EC 2.3.1.16) catalyzes linear fatty acid carbon chain shortening during β-oxidation and together with CoA catabolizes 3-ketoacyl-CoA into acetyl-CoA and acyl-CoA (Lee et al., 2009). Each oxidation cycle was found to release one acetyl-CoA molecule until the fatty acid was completely converted into acetyl-CoA (Infant et al., 2021). *POT1* deletion was found to induce abnormal growth morphology (van Roermund et al., 2022) and a significant decrease in intracellular ATP content in *S. cerevisiae*, confirming that its deletion had an effect on β-oxidation blockade (Weber et al., 2020).

Three 3-ketoacyl-CoA thiolases have been identified in *Candida albicans*: Pot1p, Fox3p, and Pot13p. Pot1p and Fox3p share high homology with the *S. cerevisiae* Pot1p (Otzen et al., 2013).

## 3 Enzymes and regulatory genes indirectly related to β-oxidation

### 3.1 Peroxisome regulatory proteins

In addition to the key enzymes directly involved in β-oxidation in *S. cerevisiae*, fatty acid metabolism in the organism also involves peroxisome regulatory proteins. Pxa1p/Pxa2p is a protein complex located on the peroxisome membrane, the primary function of which is to transport long-chain ester acyl-CoA activated in the cytoplasm to peroxisomes for β-oxidation (Baker et al., 2015). Pxa1p/Pxa2p controls the balance between internal and external acyl-CoA and fatty acid levels of peroxidase, and regulates acetyl-CoA transport according to cellular demand for acetyl-CoA (van Roermund et al., 2021).

The *PEX* gene family encodes a variety of specific peroxisome-associated proteins (Nuttall et al., 2011) and regulates various biochemical reactions in peroxisomes (Table 1). Pex11p, located on the peroxisome membrane, participates in the transportation of medium-chain fatty acids from the cytoplasmic matrix to peroxisomes. During the process of β-oxidation transport, medium-chain fatty acids are first transported to peroxisome by Pex11p, are activated by Faa2p to medium-chain fatty acyl-CoA, and then undergo β-oxidation.

The number, size, and function of peroxisomes are closely related, and the larger the peroxisomes, the stronger the β-oxidation function, but the number will be smaller. The Pex11p family is involved in the regulation of peroxisome proliferation and size. However, the exact function of Pex11p is unclear. Some researchers believe that the deletion of *PEX11* may induce decreased peroxisome function and the inability to use fatty acids as carbon source in *S. cerevisiae*, and that its overexpression may induce the formation of numerous peroxisomes with significantly reduced volumes (Huber et al., 2012). Other researchers hold a different view, suggesting that *PEX11* deletion induces the formation of large peroxisomes in *S. cerevisiae* (Van Roermund et al., 2000). Through extensive research on the regulation of peroxisome proliferation, in addition to Pex11p, several *PEX11* isoenzymes, such as Pex25p, Pex27p, Pex28p, Pex29p, Pex23p, Pex31p, and Pex32p, have been identified (Mast et al., 2016).

Aside from their involvement in the regulation of peroxisome body proliferation in *S. cerevisiae*, multiple *PEX* genes (*PEX3, PEX19 PEX5, PEX22, PEX1, and PEX6*) have been found to participate in protein transport and positioning in the peroxisome enzyme body; their specific functions are shown in Table 2.

**Table 2:** Peroxisome (PEX) gene family and their functions in *Saccharomyces cerevisiae*.

Function	PEX gene	Introduction	References
Associated with peroxisome membrane formation	*PEX3* & *PEX19*	One of the main functions of interacting Pex19p and Pex3p is to transport peroxisome membrane proteins (PMPs) through the endoplasmic reticulum (ER) in a Pex3p- and Pex19p-dependent manner	Veenhuis and Van Der Klei (2014)
	*PEX36*	Pex36p, a functional homolog of mammalian PEX16, participates in ER-to-peroxisome membrane protein transport	Farré et al. (2017)
Identifying PTS	*PEX5*	C-terminal contains six *TPR* domains, which are essential for binding with *PTS1*	DeLoache et al. (2016)
	*PEX7-PEX20*	PTS2 receptors	Gaussmann et al. (2021)
	*PEX4*	Pex4p is a ubiquitin-binding enzyme (UBC) involved in the circulation of PTS receptors in the cytoplasm. Its membrane anchor, Pex22p, interacts with the complexes, Pex1p and Pex6p, which contain adenosine triphosphate enzymes (AAA ATPase) associated with various cellular activities, and their membrane anchor, Pex15p	Judy et al. (2022)
Participates in peroxisome proliferation	*PEX11*	Pex11p regulates peroxisome number and volume, as well as the metabolic activities of related membrane proteins during the fatty acid degradation process, and is responsible for transporting medium-chain fatty acyl-CoA into peroxisomes	Kiel et al. (2006)
	*PEX25*	*De novo* peroxisome growth and membrane elongation	
	*PEX27*	Is a peripheral peroxisome membrane protein involved in regulating peroxisome size and number, and interacts with its homologous protein, Pex25p. *PEX25*, or *PEX27* overexpression in *S. cerevisiae* induces peroxisome proliferation. In addition, mutants missing either of these genes exhibit fewer but larger organelles	
	*PEX23*	Pex23p positively regulates peroxisome size	
	*PEX31* & *PEX32*	Pex31p and Pex32p negatively regulate peroxisome size	
	*PEX28* & *PEX29*	Cells missing one or both of the genes, *Pex28p* and *Pex29p*, exhibit an increased number of small peroxisomes that tend to aggregate	Mast et al. (2016)

Peroxisome-related proteins (Glover et al., 1994), which include matrix and membrane proteins (Neuberger et al., 2003), have a peroxisome localization signal (PTS sequence S/A-K/R-L/M, R-L/I-X(5)-HL). These PTS-targeted sequences include PTS1, PTS2, and PEX19BS. Matrix proteins contain the PTS1 or PTS2 sequences, which are transferred to the peroxisome matrix through binding to the cytoplasmic receptors, *PEX5* or *PEX7*. Peroxisome membrane proteins, which have the PEX19BS signal, bind to *PEX19* membrane protein receptors and are transferred to the peroxisome membrane.

### 3.2 Fatty acyl-CoA thioesterase

The acyl-CoA thioesterase, Pte1p, which is the only acyl-CoA hydrolase identified in *S. cerevisiae* (Ntamack et al., 2009), is localized in the peroxisome matrix and participates in the hydrolysis of acyl-CoA, with free fatty acid release (Caswell et al., 2022). PTE1 is expressed exogenously in *E. coli*; a wide range of substrates have been identified for the enzyme, including long-chain (C14–C18), medium-chain (C8–C12), and short-chain (C4–C6) ester acyl-CoAs (Ntamack et al., 2009). Pte1p is more active on short- and medium-chain acyl-CoA than on long-chain acyl-CoA; these include C4, C6, C10, and C12 fatty acids. Furthermore, it has a predilection for unsaturated enoyl-CoA (Kawaguchi et al., 2021).

Four acyl-CoA thioesterases were identified in mice (Table 3). *ACOT3* and *ACOT5* are long- and medium-chain acyl-CoA thioesterases, respectively (Chinopoulos, 2021). *ACOT8* has broad substrate specificity and hydrolyzes straight and branched ester acyl-CoAs. It has high homology with *S. cerevisiae PTE1*. Acot4p is a succinyl-CoA thioesterase (Tillander et al., 2017) (Table 3).

**Table 3:** Substrate specificity of different ester acyl-CoA thioesterase species.

Thioesterase species	Specific substrate	Thioesterase source	References
Pte1p	Long-chain (C14–C18), medium-chain (C8–C12), short-chain (C4–C6) ester acyl-CoAs; Pte1p exhibits higher activity on short- and medium-chain acyl-Coacyl-CoAs, with a predilection for unsaturated enoyl-CoA	*Saccharomyces cerevisiae*	Lian and Zhao (2015)
Acot4p	Succinyl-CoA	Mice	Tillander et al. (2017)
Acot3p	Long-chain (C14–C18) acetyl-CoA		Chinopoulos (2021)
Acot5p	Medium-chain (C8–C12) acetyl-CoA		
Acot8p	With a broad substrate specificity, Acot8p hydrolyzes straight- and branched-chain ester acyl-CoA, dicarboxyl-CoA, and bile acid-CoA		

Thioesterase is a key enzyme required for the β-oxidation cycle and plays an important role in maintaining the balance of free fatty acid and coenzyme A levels. It requires five essential enzymes (CAB1–CAB5) to catalyze a five-step reaction synthesis, which can be used to synthesize acetyl-CoA or enter other reaction pathways (Nielsen, 2014). The coenzyme A released by thioesterase can enter the next round of the β-oxidation cycle, and acetyl-CoA can also be synthesized into other reaction pathways, which is conducive to the intracellular circulation of coenzyme A (Zhang et al., 2021).

### 3.3 Other β-oxidizing coenzymes

In addition to the aforementioned key enzymes and regulatory genes involved in β-oxidation, some unsaturated fatty acids can enter the β-oxidation pathway only by means of auxiliary enzymes (Baker et al., 2015). When acyl-CoA substrates cannot be recognized by the key β-oxidation enzyme, Pox2p, a series of auxiliary enzymes are required to transform the substrates into suitable forms for β-oxidation. *SPS19* encodes peroxisome 2,4-dienoyl-CoA reductase, which metabolizes 2,4-dienoyl-CoA in the peroxisome to 3-enoyl-CoA in a NADPH-dependent process (Robert et al., 2005). The dodecenoyl-CoA isomerase, Eci1p, also known as ∆3(cis), ∆2(trans)-enoyl-CoA isomerase, which exhibits elevated transcription levels in oleic acid-containing media, can convert 3-enoyl-CoA into trans-2-enoyl-CoA. It is a member of the hydratase/isomerase protein family and a hexameric enzyme comprising six identical 32 kDa subunits with 280 residues each (Mursula et al., 2000; Ntamack et al., 2009).

The beta-oxidation process also requires the participation of cofactors, such as ATP and CoA, necessitating the involvement of a series of key cofactor transport-associated enzymes. (Ant1p) YPR128cp is a peroxisome ATP/AMP transporter and is necessary for peroxisome proliferation. This protein is localized in the peroxisome membrane and belongs to a subgroup of *S. cerevisiae* medium-chain fatty acid ATP/AMP transporters (El Moualij et al., 1997). YPR128cp was found to show the highest homology with *C. albicans PMP47*, *Plasmodium falciparum* adenine nucleotide translocase, and homo *PMP34* (Van Roermund et al., 2001).

During the process of fatty acid degradation, H_2_O_2_ produced is degraded by catalase Cta1p. When the acyl-CoA oxidase, Fox1p, oxidizes acyl-CoA to trans-2-enoyl-CoA, H_2_O_2_ is degraded by Cta1p, thereby preventing cellular H_2_O_2_ toxicity (Lefevre et al., 2013; Klug and Daum, 2014).

## 4 Anabolic pathways of lipids by usage of fatty acid

### 4.1 Synthesis of neutral lipids in *Saccharomyces cerevisiae*

In *S. cerevisiae*, aside from participating in the β-oxidation metabolic pathway, fatty acids can also be used as precursors for the synthesis of lipid substances (Yadav et al., 2018), providing material for cell membrane synthesis and allowing for the accumulation of intracellular fats (Gossing et al., 2018). When it comes to synthesizing products such as ceramides or other fatty acid derivatives, *Yarrowia lipolytica* has a great advantage over *S. cerevisiae* in terms of yield. However, *Saccharomyces cerevisiae*, as a gene model strain, is a good research object, which can provide metabolic pathway modification ideas for other *oil-producing yeasts* (Su et al., 2023).

**Figure 2:**
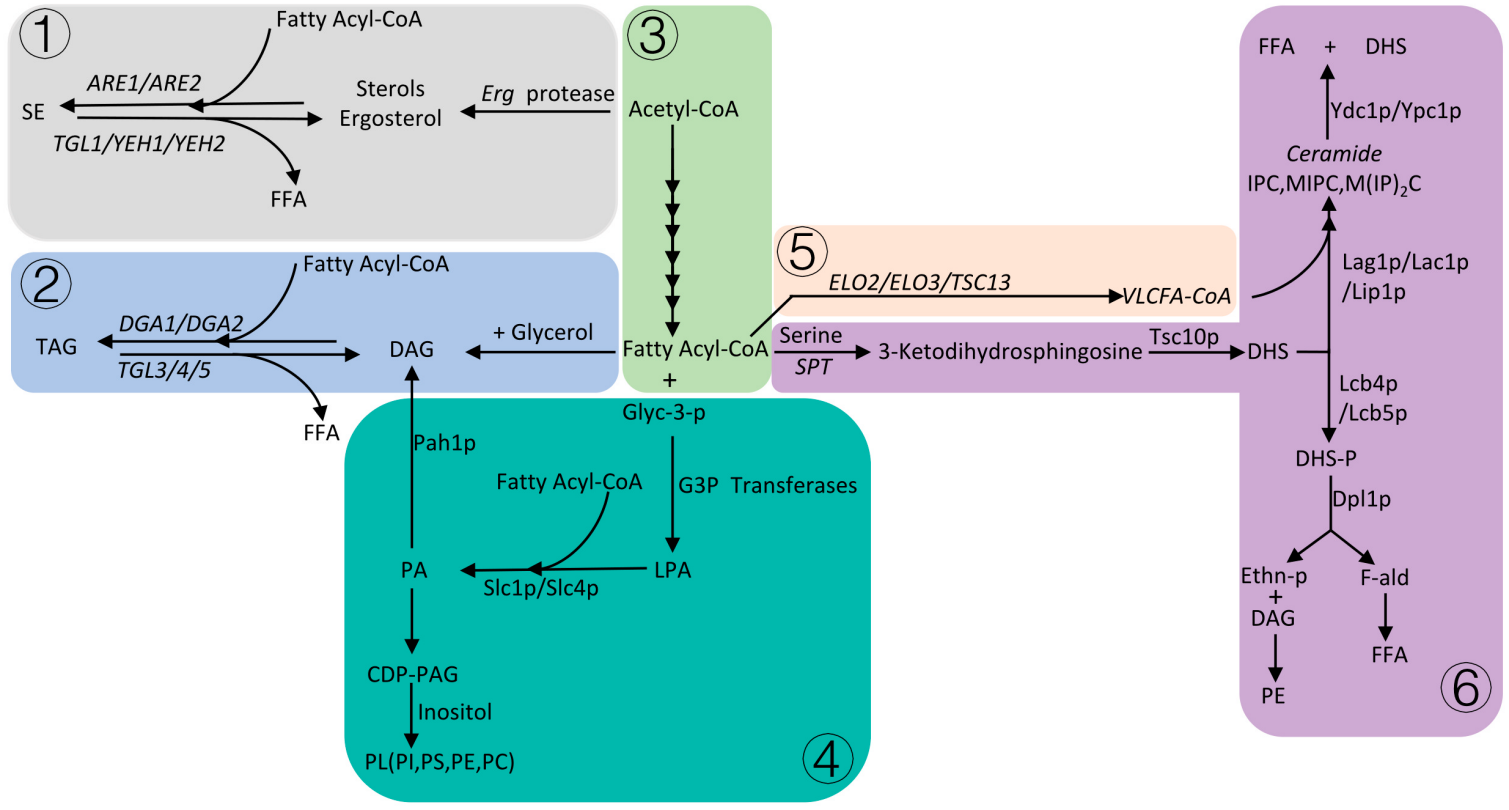
Please refer to Section 5 for details, the gray area ① is the formation process of sterol esters, the light blue area ② is the process of triglyceride formation, the light green area ③ is the abbreviation of fatty acid synthesis, the dark green area ④ is the phospholipid formation process, the light orange area ⑤ is the process of fatty acid elongation, and the purple area ⑥ is the sphingolipid anabolic process.

There are three main fatty acid derivatives of *S. cerevisiae*, which are phospholipids, sphingolipids, and neutral lipids. Neutral lipids are mainly composed of sterols and triacylglycerol. Among them, phospholipids, sterols and sphingolipids are important components of cell membranes (Lv et al., 2023). When the supply of carbon sources in *S. cerevisiae* is insufficient, neutral lipids stored in the cell are degraded to produce FFA (Free long-chain fatty acids). FFA is activated to form fatty acyl-CoA, which enters β-oxidation to provide energy to cells (Figure 2).

In *S. cerevisiae*, sterols are necessary for the integrity of cell membranes, and maintain the integrity, fluidity, and permeability of the membrane structure (Jordá and Puig, 2020). The synthesis of sterols occurs mainly in the endoplasmic reticulum, and the end product of the synthesis pathway is ergosterol (Khatri and Choudhary, 2023). Ergosterol starts from acetyl-CoA and is synthesized by nearly 30 proteases, ergosterol esters/sterol esters (SE) are formed by the action of fatty acyl-CoA and ergosterol/sterols under the action of sterol acyltransferases (ARE1/ARE2) (Koch et al., 2014). The balanced regulation of sterol synthesis pathways in yeast is very important, and excess sterols can cause toxicity to cells. At this point, cells induce proteasomal degradation of HMG-CoA reductase (HMGR), resulting in reduced sterol synthesis (Wang et al., 2023). The amount of sterols in yeast is very low, the rate of sterolipid synthesis is relatively stable during the logarithmic phase of yeast growth, and sterolipid synthesis significantly increases when yeast growth enters the stationary phase (Alvarez-Vasquez et al., 2011), under aerobic conditions, yeast is prone to produce excess sterols. In order to reduce the toxicity caused by excess sterols, sterols can be transferred to the plasma membrane in the form of sterol acetate and then secreted into culture media, or sterols can be esterified and stored in lipid droplets (LD) inside the cell (Pagac et al., 2011).

Triacylglycerol (TAG) is formed by the glycerol backbone and fatty acyl-CoA under the action of diacylglycerol O-acyltransferase (DGA1/LRO1), which can be used as energy storage and as a component of cell membranes (Khatri and Choudhary, 2023). When cells are starved, sterol esterases (TGL1/YEH1/YEH2) degrade sterol esters stored in LD to free fatty acids, and difunctional triglyceride lipase/lysophosphatidyletha nolamine acyltransferase (TGL3/4/5, ARY1) degrades triacylglycerol to FFA, which participates in β-oxidative degradation to acetyl-CoA for cellular energy (Ke et al., 2023).

### 4.2 Synthesis of phospholipids in *Saccharomyces cerevisiae*

The phospholipids (PL) in *S. cerevisiae* mainly include phosphatidylcholine (PC), phosphatidylethanolamine (PE), phosphatidylinositol (PI), and phosphatidylserine (PS). Phosphatidic acid (PA) is a central metabolite in the synthesis of PL (Engelhardt et al., 2023), and a series of synthesis occurs in the endoplasmic reticulum. First, fatty acyl-CoA and glycerol-3-phosphate (Glyc-3-P) synthesized lysophosphatidic acid (LPA) through G3P acyltransferases encoded by SCT1 (GAT2) and GPT2 (GAT1) in the endoplasmic reticulum (Yasukawa et al., 2023), and LPA and fatty acyl-CoA formed PA through the LPA acyltransferase Slc1p/Slc4p in the endoplasmic reticulum, and then formed cytidine diphosphate diacylglycerol (CDP-DAG). CDP-DAG is a precursor for the synthesis of PL, which forms PI, PS, PE, PC under the action of celluloalcohol (Matos et al., 2023). PA can be cleaved into diacylglycerol (DAG) by phospholipidase phosphatase Pah1p, which is then combined with fatty acyl-CoA to act as a storage lipid in cells (Speer et al., 2024). Although the composition of organelle membranes varies, film-forming PL is necessary for the formation of various organelles. The expression of synthetic PL genes was affected by various growth conditions such as temperature, growth medium, pH and nutrients, but PA, as the central metabolite of synthetic PL, had the greatest impact on PL. PA is regulated by a variety of enzymes, the most important being PA phosphatase Pah1p and DG kinase Dgk1p, overexpression of *DGK1* or knockout of *PAH1* can enhance PA synthesis (Khaddaj et al., 2023).

### 4.3 Synthesis of sphingolipids in *Saccharomyces cerevisiae*

Sphingolipids (SL) are mainly synthesized in the endoplasmic reticulum and are transported to the plasma membrane or other organelles after synthesis (Klug and Daum, 2014). The synthesis of complex ceramides begins with fatty acyl-CoA (mainly palmitoyl-CoA or stearoyl-CoA) and condenses with serine in a serine palmitoyltransferase (SPT) complex to synthesize 3-ketodihydrosphingosine. 3-Ketodihydrosphingosine can be transformed into dihydrosphingosine (DHS) by the 3-ketosphingosine reductase (Tsc10p) (Schäfer et al., 2023), and one of the pathways for DHS is to bind to VLCFA (C20–C26) (Very-long-chain fatty acids) acyl-CoA (extended by the enzymes encoded by the three genes ELO2, ELO3, and TSC13) in response to the ceramide synthase complex (Lag1p, Lac1p, and Lip1p), and then undergo a series of reactions to produce ceramides, including inositol phosphoceramide (IPC)/Mannositide phosphoceramide (MIPC)/mannosyldiinositide phosphoceramide (M(IP)_2_C) (Willet et al., 2023). The alkaline dihydrofibrinoenzymes Ydc1p and Ypc1p are responsible for catalyzing ceramides into FFA and DHS. The other route is phosphorylation catalyzed by Lcb4p and Lcb5p to form dihydrosphingosine phosphate (DHS-P), which is cleaved by the DHS phospholyase Dpl1p to synthesis ethanolamine. Phosphate (Ethn-P) and fatty aldehyde (F-ald). Ethn-P can synthesize PE with DAG through a series of reactions, F-ald is metabolized to FFA. FFA is activated in the cytosol and then enters the β-oxidative degradation for energy.

## 5 Application of the regulation of fatty acid metabolism for fatty acid synthesis

### 5.1 Synthesis of fatty acids and fatty alcohols in *Saccharomyces cerevisiae*

As a single cell-type strain, *S. cerevisiae* exhibits good biosafety and robustness. Compared with strains that are harmful to humans, *S. cerevisiae* is certified as a GRAS (Generally Recognized as Safe) strain (Lian et al., 2018), produces fatty acids and products that are safer, the comparison of oil-producing yeast to *S. cerevisiae* is shown in Table 4. Modifying the fatty acid metabolic pathway is of great value in improving the synthesis of fatty acids and their derivatives.

**Table 4:** Comparison of the advantages and disadvantages of *Saccharomyces cerevisiae* and *Yarrowia lipolytica*.

Comparison of advantages and disadvantages	*Yarrowia lipolytica*	*Saccharomyces cerevisiae*	References
Synthesis of polyunsaturated fatty acids	The oil yield is high, about 77%	The oil yield is low, about 15%	Zhang et al. (2014), Li et al. (2006)
Tolerance to the environment	High tolerance to organic acids and low pH	High tolerance to organic solvents (ethanol) and harsh culture conditions (oxidative stress, high concentrations of NaCl)	Auesukaree et al. (2009), Cui et al. (2017)
Efficiency of gene editing tools	Low efficiency	One of the most efficiently and well-researched strains	Lian et al. (2018)
Cell growth rate	Low, specific growth rate < 0.2/h	Higher, the specific growth rate < 0.4/h	Moeller et al. (2007)
Synthetic fatty acid ethyl esters	High fatty acid ethyl ester titer, 0.14 titer (g/L)	Fatty acid ethyl ester titer low, 0.034 titer (g/L)	Shi et al. (2014), Xu et al. (2016)
The extent to which cells utilize organic matter	Cheap hydrophobic substrates can be effectively utilized, and unsaturated fatty acids have less influence on them	Unsaturated fatty acids significantly affect the resistance of cells and damage the cell membrane	Ruenwai et al. (2011)
Genome sequencing	Part of the genome sequence has been made public	The sequencing of the entire genome has been published and can be used to study the mechanisms of oil production in eukaryotic systems	Wynn et al. (1999)

Medium-chain fatty acids contain 8–12 carbon atoms. Compared with long-chain fatty acids, esters related to medium-chain fatty acids have an aroma and can be combined with other substances to form fuels; polymers can also be synthesized in materials, and are used in fields such as health care drugs, cosmetics, and foods to speed up metabolism (Garces Daza et al., 2023). The accumulation of saturated long-chain fatty acids under fermentation conditions induces the release of medium-chain acyl-CoA by fatty acid synthase (FAS) complexes (Mbuyane et al., 2022). To induce the production of more medium-chain fatty acids in *S. cerevisiae*, Pox1p was knocked down, while Pox2p (from *Y. lipolytica*) and carnitine O-octanoyl transferase Crotp overexpression was induced; this resulted in the secretion and accumulation of medium-chain fatty acids in the medium, with a corresponding increase in medium-chain fatty content. The intracellular medium-chain fatty acid (C8:0, C10:0, and C12:0) titers in *Δ pox1* [*pox2*+] 和 *Δpox1* [*pox2*+,*crot*+] train were 2.26 and 1.87 fold higher than those in WT strain, and their extracellular titers in the modified strain were 3.29 and 3.34 fold higher than those in the WT strain (Chen et al., 2014a).

Long-chain fatty acids are components of cell membranes, which have strong inhibitory properties against foodborne pathogens such as Salmonella, can effectively regulate intestinal health after use, and are widely used in medical and food fields (Borreby et al., 2023). Currently, they are typically obtained from plants, animals, and petroleum. Producing them using microorganisms is a renewable approach. Fatty acids (16C–18C) are synthesized in yeast from acetyl-CoA by the action of type I FAS (Nancolas et al., 2017). By inducing the overexpression of the acetyl-CoA carboxylase, Acc1p, the fatty acid synthase, RtFAS (*Rhodosporidium toruloides* FAS), and *E. coli* thioesterase, *TesA*, in combination with the knock out of the two main acyl-CoA synthetase genes, *FAA1* and *FAA4*, as well as that of the torulosis acyl-CoA oxidase gene, *POX1*, fatty acid production was significantly improved to 10.4 g/L in *S. cerevisiae* (Zhou et al., 2016).

Genetic engineering is used to regulate cell metabolic pathways to increase the accumulation of FFA in yeast cells (Zhang et al., 2020). Firstly, the conversion of FFA to acyl-CoA was blocked by knocking out *FAA1* and *FAA4* genes (Chen et al., 2014b). A truncated version of acyl-CoA thioesterase *ACOT5* (Acot5s), which encodes *Mus musculus* peroxisomal acyl-CoA thioesterase 5, is then expressed in the cytoplasm, enhancing the conversion of Acyl-CoA to FFA. Strains with double deletion of *FAA1* and *FAA4* genes and overexpression of thioesterase ACOT5s increased FFA levels nearly 6.43 times in the medium, and the proportion of unsaturated fatty acids (UFA) was higher. In addition, the expression levels of genes associated with fatty acid synthesis, *ACC1, FAS1, FAS2*, and *OLE1*, were also increased in RT-PCR analysis. These results suggest that FFA accumulation in yeast cells can be increased by regulating acyl-coA metabolism.

To address the imbalance of redox factors in FFA production (acetyl-CoA supply, low NADH/NAD ratio), FFA-producing strains with four gene deletions (*faa1, Δfaa4, Δpox1, Δfaa4*) were used. *ΔHFD1* as a chassis cell (Zhou et al., 2016) knocked out two endogenous NAD-dependent glycerol-3-phosphate dehydrogenase genes GPD1 and GPD2 (to reduce redox imbalance during glucose synthesis of fatty acids). Overexpression of NADP-dependent glyceraldehyde-3-phosphate dehydrogenase (GapN) in heterostreptococcus mutants (Zhang et al., 2016) balanced the redox factors of NAD and NADH in the cytoplasm. Overexpression of the bacterial pyruvate dehydrogenase PDH complex (increased supply of acetyl-CoA) significantly increased cell growth, decreased ethanol consumption, and decreased glycerol accumulation. The PDH7 strain produced 840.5 mg/L FFA at 72 h, and the final experimental results showed that the optimal fatty acid producing strain PDH7 produced 840.5 mg/L FFAs in the shaker, which was 2.08% higher than that of the control strain. At the same time, the distribution ratios of saturated and unsaturated FFA (C16 and C18) also increased (Zhang et al., 2020).

The synthesis of fatty alcohols using microorganisms has several applications (Hu et al., 2020). Biosynthetic natural higher fatty alcohols (medium-chain fatty alcohols) can be used as the basic raw materials for fine chemical products such as cosmetics, detergents, surfactants, plastic plasticization, etc., and medium-chain fatty alcohols have high energy density properties and can be used as alternative fuels to traditional diesel. Most of the fatty alcohols currently used are extracted from petroleum or produced from fatty acids extracted from vegetable oils (oilseeds). Biosynthetic fatty alcohols are sustainable. Fatty acids and fatty aldehyde intermediates are usually used for synthesis reactions (Yaguchi et al., 2018). Fatty alcohol synthesis in wild-type *S. cerevisiae* is completed in the cytoplasm. After fatty acids are activated by fatty acyl-CoA synthetase in the cytoplasm, a portion of the activated fatty acids is reduced to form fatty aldehydes and alcohols. By knocking out the fatty alcohol oxidation gene, *HFD1*, and inducing the overexpression of alcohol dehydrogenase and aldehyde reductase, the fatty alcohol yield can reach 1.5 g/L (Zhou et al., 2016). To produce more medium-chain fatty alcohols in peroxisomes, the overexpression of fatty acyl-CoA reductase (TaFAR), which utilizes medium-chain fatty acyl-CoA produced in peroxisomes to produce medium-chain fatty alcohols, but no 1-tetradecanool production was found in the assay results. On this basis, acetyl-CoA carboxylase (Acc1p) and ATP-dependent citrate lyase (Aclp) overexpression was induced in *S. cerevisiae* to increase fatty acid titers in the cytoplasm; finally, the medium-chain fatty alcohol titer in the modified strain was increased to 1.3 g/L (Sheng et al., 2016).

### 5.2 Establishment of a β-oxidation reversal reaction for the synthesis of fatty acids and their derivatives

The formation of fatty acids and their derivatives in *S. cerevisiae* using acetyl-CoA is catalyzed by fatty acid synthetase, and this requires the use of ATP and ACP (acyl carrier protein), as well as the reducing power of NAPDH. Compared with this pathway, reversing β-oxidation requires less energy and has greater potential in fatty acid synthesis.

Thus, induction in the cytoplasm can reverse the overexpression of core enzymes of β-oxidative function (Lian and Zhao, 2015), including acetyl-CoA synthase (ACS), β-ketoacyl-CoA synthase (KS), β-ketoacyl-CoA reductase (KR), β-hydroxyacyl-CoA dehydrase (HTD), trans-2-ene-CoA reductase (TER), and thioesterase (CpFatB1), and can increase the production of short- and medium-chain fatty acids. “N-butanol” accumulation was induced through the overexpression of CoA-acylated aldehyde dehydrogenase (EcEutE) (Eriksen et al., 2014) and clostridium acetylbutanol dehydrogenase (CaBdhB). In addition, acyl-CoA:ethanol O-acyltransferase (EEB1/EHT1) overexpression was induced to successfully synthesize fatty acid ethyl esters (Strijbis and Distel, 2010).

**Figure 3:**
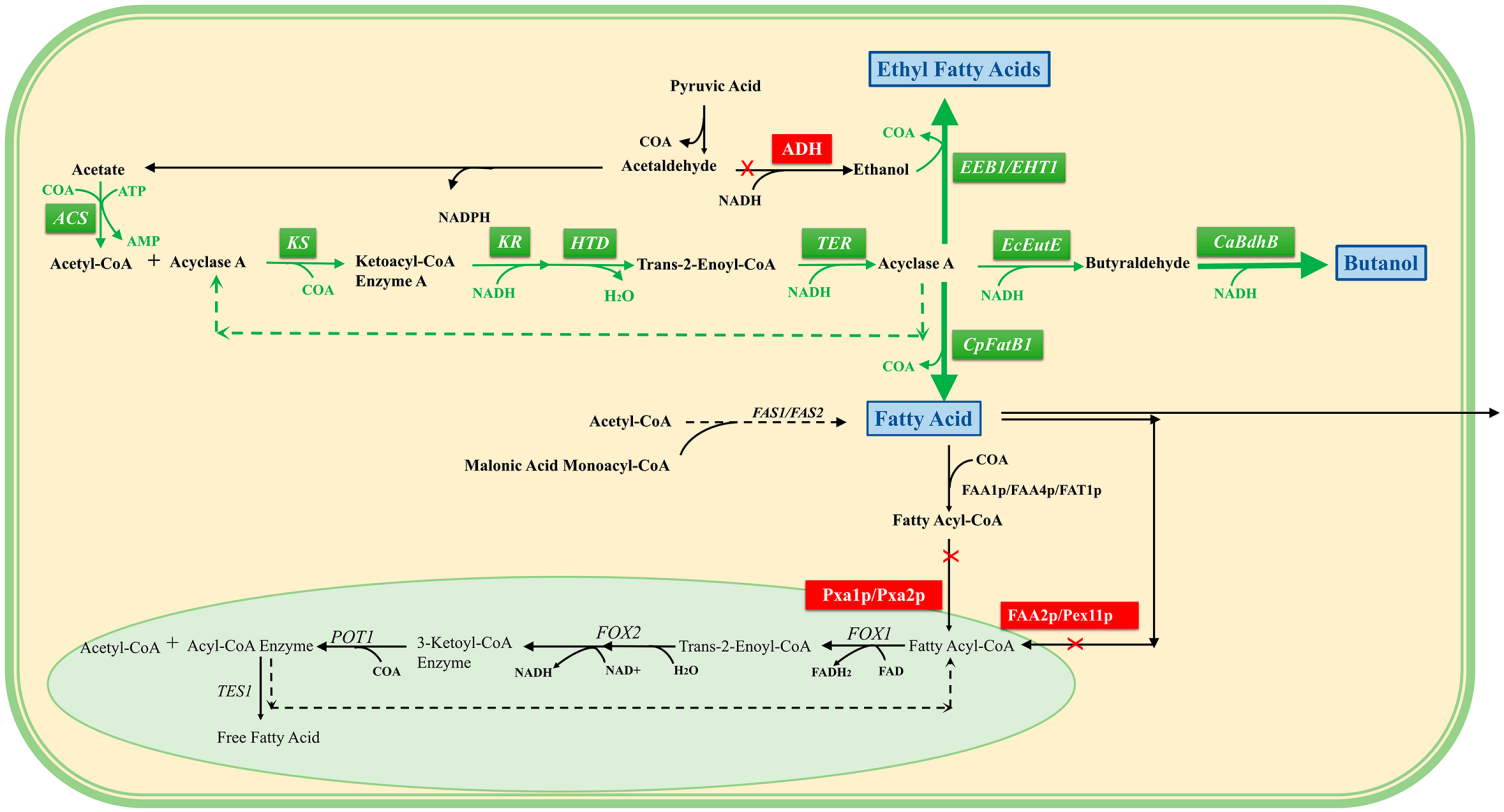
Reversal of the β-oxidation pathway in the cytosol. A pale green background indicates peroxisomes, and a pale yellow background indicates cytoplasm. The green box is the overexpressed reverse β-oxidation-related enzyme, the blue box is the product, the red box is the blocking pathway, the black arrow is the direction of the intracellular original reaction, and the green arrow is the direction of the overexpression construction pathway. It can be seen that the intermediate products of the reversed β-oxidation pathway constructed in the cytosol are consistent with the β-oxidation intermediate products in the peroxisome, and finally three target products can be obtained.

To carry out the β-oxidation reversal reaction more efficiently, the supply mechanism of redox cofactors in the β-oxidation process was explored, and MCFA was used as the product to optimize the conditions. Stoichiometric analysis of the β-oxidation pathway showed that NADH was the key factor affecting the oxidation efficiency of β, and the highest titer of MCFA reached 4.7 g/L after strengthening the supply of acetyl-CoA and NADH, overexpressing the formate dehydrogenase of *Candida tropicalis*, and optimizing the acetate reassimilation pathway (Wu et al., 2017). (This section refers to Figure 3).

## 6 Future perspectives

In recent years, the demand for fatty acids and their derivatives in the medical, health, cosmetic, and food sectors has increased. The efficient production of fatty acids by microorganisms is a promising approach (Saravanan et al., 2022). *Saccharomyces cerevisiae* is a preferred host for the synthesis of high-value products. By controlling the β-oxidation process, as well as other metabolic pathways, *S. cerevisiae* can be modified to be a novel platform for the production and accumulation of fatty acids and their derivatives (Akşit and Van Der Klei, 2018).

Taking advantage of the similarity of fatty acid synthesis pathways in *oil-producing yeast* and *non-oil-producing yeast*, the content of specific fatty acids is increased by genetic engineering of *S. cerevisiae* to overexpress heterologous genes, thereby enriching the potential products of *S.cerevisiae* (Biernat et al., 2023).

At present, the fatty acid synthesis of *S. cerevisiae* is dominated by long-chain fatty acids C16:0, C16:1, C18:0 and C18:1, because the content of C16/C18 in the cell membrane of *S. cerevisiae* is higher (Hashem et al., 2021), which is more conducive to the synthesis of fatty acid derivatives. *Saccharomyces cerevisiae* has many potential products of fatty acids, aldehydes, alcohols and alkanes (Kim et al., 2021), but the tolerance of these potential products needs to be improved. For example, lipophilic medium-chain fatty acids tend to damage the plasma membrane of *S. cerevisiae*, reducing the plasma membrane’s ability to resist the external environment (Liu et al., 2022). In a study, it was found that the production of medium-chain fatty acids in wild-type *S. cerevisiae* was low, and exogenous medium-chain fatty acids had an inhibitory effect on the growth of *S. cerevisiae*; The accumulation of alkanes through overexpression of thioesterase can alleviate the damage of medium-chain fatty acids to cell membranes (Zhu et al., 2017). Medium chain fatty acids and their derivatives can be used as potential products of *Saccharomyces cerevisiae* and have great room for improvement. In the future, transcriptome analysis can be used to screen key genes for medium-chain fatty acid tolerance in *Saccharomyces cerevisiae*, and then use the advanced gene editing technology CRISPR-Cas9 to modify related metabolic pathways in *Saccharomyces cerevisiae*. In future fermentation production, fed-batch strategies such as *in-situ* adsorption, substrate product separation, and substrate embedding techniques can be carried out according to different substrates to minimize the damage of the product to cells.

Peroxisomes are a promising compartment for regulating fatty acid degradation. Knockdown of peroxisome regulatory genes *PEX11*, *PEX25*, and *PEX27* showed that changes in the number of peroxisomes had an impact on the longevity of yeast (Deb and Nagotu, 2023). During β-oxidation, energy is required for fatty acid degradation, and the energy provided by mitochondria to peroxisomes passes through the cytoplasmic matrix. How to significantly improve the β-oxidative energy supply of peroxisomes and effectively regulate the metabolic flux of NADP/NADPH and ATP/AMP transport rate is a difficult task to improve the metabolic efficiency of peroxisome, In the future, the pathway of coenzyme A in peroxisome can be explored by isotope labeling, and the regulation process of key genes or protein families on cofactor delivery can be reasoned by transcriptome analysis.

## Author contributions

ZW: Writing – original draft. CS: Writing – original draft. RW: Writing – review & editing. JS: Writing – review & editing. YZ: Writing – review & editing and software. SS: Writing – review & editing.
